# Burden and costs of migraine in a Swedish defined patient population – a questionnaire-based study

**DOI:** 10.1186/s10194-019-1015-y

**Published:** 2019-05-31

**Authors:** Frida Hjalte, Sara Olofsson, Ulf Persson, Mattias Linde

**Affiliations:** 1 The Swedish Institute for Health Economics (IHE), Lund, Sweden; 2 Department of Neuromedicine and Movement Sciences, NTNU Norwegian University of Science and Technology, Trondheim, Norway; 3 Tjörn Headache Clinic, Tjörn, Rönnäng Sweden

**Keywords:** Migraine, Cost, Work productivity, Quality of life, EQ-5D, Sweden

## Abstract

**Background:**

Migraine is a disabling, chronic neurological disease leading to severe headache episodes affecting 13.2% of the Swedish population. Migraine leads to an extensive socio-economic burden in terms of healthcare costs, reduced workforce and quality of life (QoL) but studies of the health-economic consequences in a Swedish context are lacking.

The objective of this study is to map the health-economic consequences of migraine in a defined patient population in terms of healthcare consumption, production loss and QoL in Sweden.

**Methods:**

The study is based on data from a web-based survey to members in the Swedish patients’ association suffering from migraine. The survey was conducted in May 2018 and included people with migraine aged 18 years or older. The survey included questions on health resource consumption, lost production resulting from migraine-related absenteeism and presenteeism, and QoL as measured by the EuroQol 5 dimensions questionnaire (EQ-5D-5 L) and the Headache Impact Test (HIT-6). The results are presented in yearly costs per patient and losses in quality adjusted life years (QALYs).

**Results:**

The results are based on answers from 630 individuals with migraine and are presented by number of migraine days per month. The total cost per patient and year increased with the number of migraine days per month (*p* < 0.001) and varied between approximately €5000 for those with less than 3 migraine days per month and €24,000 per year for those with 21–28 migraine days per month. Production loss represented the main part of the costs, approximately 80%. The average loss in QALYs per year also increased with the monthly number of migraine days (*p* = 0.023).

**Conclusions:**

Migraine leads to significant societal costs and loss of quality of life. There appears to be an unmet need and a potential for both cost savings and QoL benefits connected with a reduction in the number of migraine days.

**Electronic supplementary material:**

The online version of this article (10.1186/s10194-019-1015-y) contains supplementary material, which is available to authorized users.

## Background

Migraine is a disabling, chronic neurological disease characterized by severe headache episodes with throbbing pain in combination with nausea, vomiting, light sensitivity (photophobia) and sound sensitivity (phonophobia) [1]. 13.2% of the Swedish population suffers from migraine and the disease is more common among women (16.7%) than among men (9.5%) [2]. In a previous study of the disease burden of migraine in Sweden, people with migraine had on average 1.3 migraine episodes per month, which lasted for 19 h on average [3]. A majority (65%) reported absence from school or work in the past year due to migraine and over 50% reported that migraine affected leisure time, family situation and work ability.

Chronic migraine (CM) is defined in accordance with the International Classification of Headache Disorders (ICHD-3) as at least 15 headache days per month, of which at least 8 with migraine [4]. Episodic migraine can be divided into low frequency and high frequency depending on the number of headache days per month where low frequency has been defined as 1–9 days a month and high frequency as 10–14 days per month [5]. About 1–2% of the general population is estimated to have CM [6].

Migraine pharmacological treatment consists of both acute and preventive therapies. Acute drugs include paracetamol, non-steroidal anti-inflammatory drugs (NSAIDs) and triptans [7]. Preventive drug treatments include beta blockers, angiotensin-2 inhibitors, antiepileptics, antidepressants and botulinum toxin type A [7]. Preventive treatment usually does not render the patient migraine free but can lead to reduced frequency of migraine days and milder symptoms.

Evidence-based nonpharmacological therapeutic alternatives for treating migraine include nutraceuticals (e g. riboflavin), invasive and non-invasive neuromodulation and different behavioural techniques and acupuncture [8].

Several studies have shown that migraine leads to an extensive socio-economic burden in terms of direct costs, i.e. resources required for health care and treatment [9], indirect costs, i.e. the value of the lost production resulting from migraine-related absenteeism and presenteeism (reduced productivity at work related to migraine) [10, 11] and reduced quality of life (QoL) [11–15]. An earlier European study has reported yearly health care costs related to migraine between € 500 and 3700 per person depending on country and migraine frequency [9]. The average societal cost of migraine in eight European countries has previously been reported to more than € 1200 per person and year where production loss accounted for the main part [10]. Previous studies show that the disease burden varies widely between different countries. However, studies analysing health-economic consequences in a Swedish context are lacking.

The primary objective of this study was to map the health-economic consequences of migraine in a defined patient population in terms of healthcare consumption, production loss and QoL in Sweden based on a survey of people with migraine. The secondary objective is to investigate how these consequences vary with the number of days of migraine and other background characteristics.

## Method

A survey was conducted in May 2018. The Swedish patients’ association, Huvudvärksförbundet, sent out a link to a web-page with the questionnaire to all members with a registered e-mail address, a total of 2894 individuals. Two reminders were sent. Inclusion criteria were having migraine (no specific diagnosis criteria) and being 18 years or older. The respondents were not reimbursed for their participation.

The web-based questionnaire was designed to include questions about age, gender, migraine duration, migraine frequency, diagnoses, number of visits to physicians and visits to other healthcare providers due to migraine, acute drug treatment and preventive drug treatment, sickness absence and QoL. QoL was measured by the generic instrument, Euro-Qol 5 dimensions – 5 levels (EQ-5D-5 L) [16], and a disease-specific instrument, HIT-6 (Headache Impact Test) [17]. The EQ-5D is widely used to measure health-related QoL in health economics analyses. Respondents were asked to assess their current health status using EQ-5D-5 L followed by a question on whether they currently suffered from a migraine episode or not. Depending on the response, the respondent was instructed to recall their last day with/without migraine and again respond to questions on the health status of that day using EQ-5D-5 L.

The number of health care visits and the use of preventive drug treatment were asked with a 12 month recall period while acute drug treatment and sickness absence were asked with a 4 week recall period. Due to the natural course of the disease which is characterized by “ad hoc” ictal phases it was assumed that it would be more difficult for respondents to recall their long-term use of acute drug treatment as well as sickness absence beyond a 4 week recall period.

The results are presented by number of migraine days per month using the following categories; 0,^1^ 1–3, 4–5, 6–7, 8–9, 10–14, 15–20 and 21–28 days. Health care costs were calculated by multiplying the resource use reported in the survey with unit prices from a regional price list for the Southern Health Care Region in 2018 [18], which is one of the most detailed sources of information on the costs of health care resources in Sweden. Pharmaceutical costs were calculated according to AUP prices (retail/pharmacy-level prices) from Pharmaceutical Specialties in Sweden (FASS) [19]. The production loss due to short-term work absenteeism was calculated by multiplying days of sick leave by the sex- and age adjusted average wage including pay roll taxes in accordance with the human capital approach [20]. The production loss due to long-term work absenteeism was based on the assumption that respondents with migraine and long-term sick-leave would have had the same productivity as the general population (of corresponding age and sex) if they would have been free of migraine. Production loss due to reduced presenteeism was likewise calculated but multiplied with the point estimate from the VAS scale (from 0 to 10), where the respondent indicated to what extent productivity was reduced while working with migraine. The calculation of total yearly costs for acute drugs and production loss assumed that the 1-month period reported was representative for the rest of the year, thus multiplying the monthly cost with 12.

**Fig. 1:**
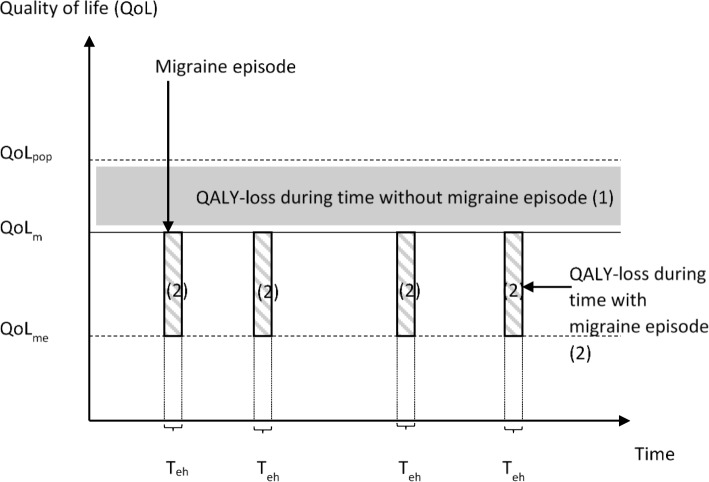
Simplified illustration of QALY loss related to migraine (QoL_pop_ = QoL for the general population; QoL_m_ = QoL at a day without a migraine episode; QoL_me_ = QoL at a day with a migraine episode; T_eh_ = time with migraine episode in terms of hours)

The loss of a Quality Adjusted Life Years (QALY) is calculated by multiplying QoL weights ​​for a specific health state by the time spent in that health state. The loss in QoL considered both the loss caused by the chronic feature of migraine (interictal phase) and the loss caused by the migraine episode (ictal phase). Figure 1 illustrates this loss of a QALY related to migraine where the grey area illustrates the loss during the interictal state and the grey striped areas represent the QALY loss during ictal phases. The QoL weights used in this study are derived from Devlin et al. 2018 [21]. The QoL weights for the general population (baseline estimates) are derived from Dolan et al. 1995 [22]. The reason for different weights is that this study used the EQ-5D-5 L while the study of the Swedish general population [23] used the EQ-5D-3 L.

Differences were assessed using t-test. A *p*-value below 0.05 was considered statistically significant.

An OLS (Ordinary Least Squares) regression was performed to identify predictors of costs and the loss of QALYs related to migraine.

All costs were calculated in Swedish krona (SEK), 2017 prices, and converted to Euros (SEK 1 = € 0.089).

Data management and statistical analyses were performed using STATA Statistical Software: Release version 14.2 College Station, Texas, USA.

Ethical approval for the study was obtained by the Regional Ethical Board in Lund in 2018 (Dnr 2018/347). Informed consent was obtained from all individuals included in the study.

## Results

Out of the 2894 individuals who received the questionnaire, 795 individuals responded and met the inclusion criteria. From those who responded 630 completed the questionnaire. The results in the study are based on the 630 answers.

Table 1 presents background characteristics of the individuals included in the study. The average age of respondents was 50 years and the majority were women (87.8%). A majority (85.2%) of respondents had suffered from migraine for over 15 years. Nearly all respondents (96.4%) had been diagnosed by a doctor and almost half (46.5%) had been diagnosed with CM.

**Table 1:** Background characteristics

	*n* = 630
Age, mean (SD)	50.1 (11.7)
Women (%)	553 (87.8)
Level of education (%)^a^	
Elementary school	15 (2.4)
Secondary school	170 (27.3)
University	406 (65.3)
Other	31 (5.0)
Occupation (%)	
Employed or self-employed	479 (76.0)
Student	11 (1.8)
Retired	78 (12.4)
Sick-leave	30 (4.8)
Unemployed	15 (2.4)
Other	17 (2.7)
Migraine duration (%)	
< 1 year	2 (0.3)
1–5 years	12 (1.9)
6–10 years	31 (4.9)
11–15 years	48 (7.6)
> 15 years	537 (85.2)
Diagnosis by doctor, *n* (% of all)^b^	607 (96.4)
Migraine with aura	202 (32.1)
Migraine without aura	282 (44.8)
Chronic migraine	293 (46.5)
Episodic migraine	46 (7.3)
Other/don’t know	91 (14.4)
Other disease, n (% of all) ^b^	361 (57.3)
Consequence of previous trauma or head surgery	23 (3.7)
Cardiovascular disease	26 (4.1)
Psychiatric disease	139 (22.1)
Sleep disorder	85 (13.5)
Allergy	122 (19.4)
Other headache diagnosis	95 (15.1)
Other	224 (35.6)
HIT-6, mean (SD)^c^	63.7 (5.5)

^a^*n* = 622, ^b^More than one diagnosis or disease possible, ^c^Scale: 36 (never problem of all dimensions) to 78 (always problem at all). 60+ corresponds to “severe impact”

**Fig. 2:**
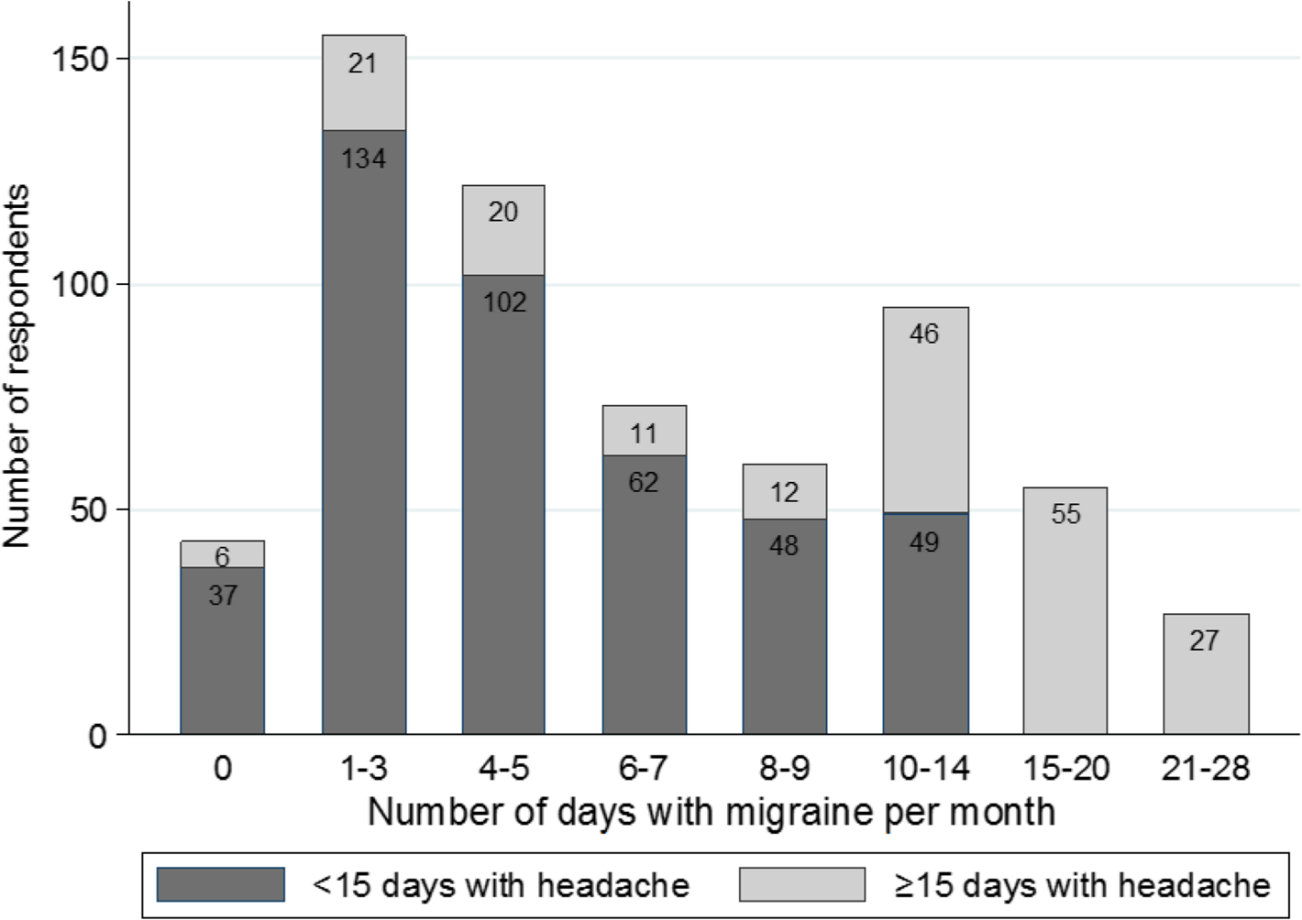
Number of respondents by number of migraine days per month divided into groups with < 15 and ≥ 15 headache days per month

Over the last 4 weeks, 6.8% of the respondents had no days of migraine, 55.6% had 1–7 days and 37.6% had more than 8 days with migraine (Fig. 2). Around 22.2% had CM based on self-reported number of migraine and headache days. Mean number of days with headache for all respondents was 12 days, including 7 days of migraine activity over the last 4 weeks. More than half of respondents had a migraine episode lasting less than 24 h. 52.9% of the respondents reported that last 4 weeks corresponded to an average month regarding migraine frequency, 30.4% reported it as a “better” month than average, and 14.3% that the last month was “worse” than an average month.

During the last 12 months, 44.8% of the respondents had visited a neurologist and 45.4% had visited a general practitioner in primary care. Almost one third of the respondents had visited a physiotherapist during the last 12 months. The most common examinations, related to migraine, were blood test and MRI (magnetic resonance imaging) (Table 2).

**Table 2:** Health care visits, examinations and drugs related to migraine during the last 12 months

Type of health care	Number of respondents (%) (*n* = 630)	Mean number of visits/examinations (SD)
Visits	57 (9)	2.8 (8.0)
Emergency room visit		
Neurologist	282 (45)	3.0 (2.2)
General practitioner, primary care	286 (45)	2.8 (2.7)
Other doctor	53 (8)	2.3 (2.0)
Nurse	75 (12)	4.1 (5.5)
Physiotherapist	184 (29)	9.6 (11.9)
Occupational therapist	24 (4)	2.9 (3.3)
Psychologist	57 (9)	6.2 (6.6)
Social worker	32 (5)	5.1 (4.5)
Other	84 (13)	8.2 (8.5)
Inpatient stay (days)	14 (2)	5.5 (5.9)
Examinations		
MRI (magnetic resonance imaging)	66 (10)	1.0 (0.5)
CT scanning (computer tomography)	39 (6)	1.0 (0.5)
ECG (Electrocardiography)	46 (7)	1.5 (1.0)
Bloodtest	147 (23)	2.2 (2.3)
Other	41 (7)	1.9 (1.4)
Drugs		
Acute drugs	588 (93)	
Preventive drugs	278 (44)	

The health care costs varied between €800 for those with less than 4 migraine days per month and up to €4000 for those who had at least 15 migraine days per month. The costs for visits to emergency rooms and inpatient days were almost exclusively accounted for by patients with migraine at least 15 days per month.

The most common acute drug was sumatriptan followed by paracetamol. The most common preventive drug was botulinum toxin type A (e.g. Botox®), used by 39.7% of respondents. 35.4% of the respondents indicated that they were not satisfied with their treatment, while 25.9% responded that they were satisfied. A majority (56.0%) had changed drug treatment or terminated treatment at least once, and 27.0% had changed drug treatment or terminated treatment at least three times.

Among all respondents with an employment or with self-employment 76.2% (*n* = 366) reported sick leave at some point during the last 12 months. On average, the respondents had reported 22 days of sick leave during the last 12 months. Eighty-nine percent (*n* = 429) stated that they worked in average 30 days while having migraine (presenteeism) during the last 12 months. In addition to the reported short-term sick leave, 17.9% of all respondents reported long-term sick leave. The total production loss increased with the number of migraine days.

**Fig. 3:**
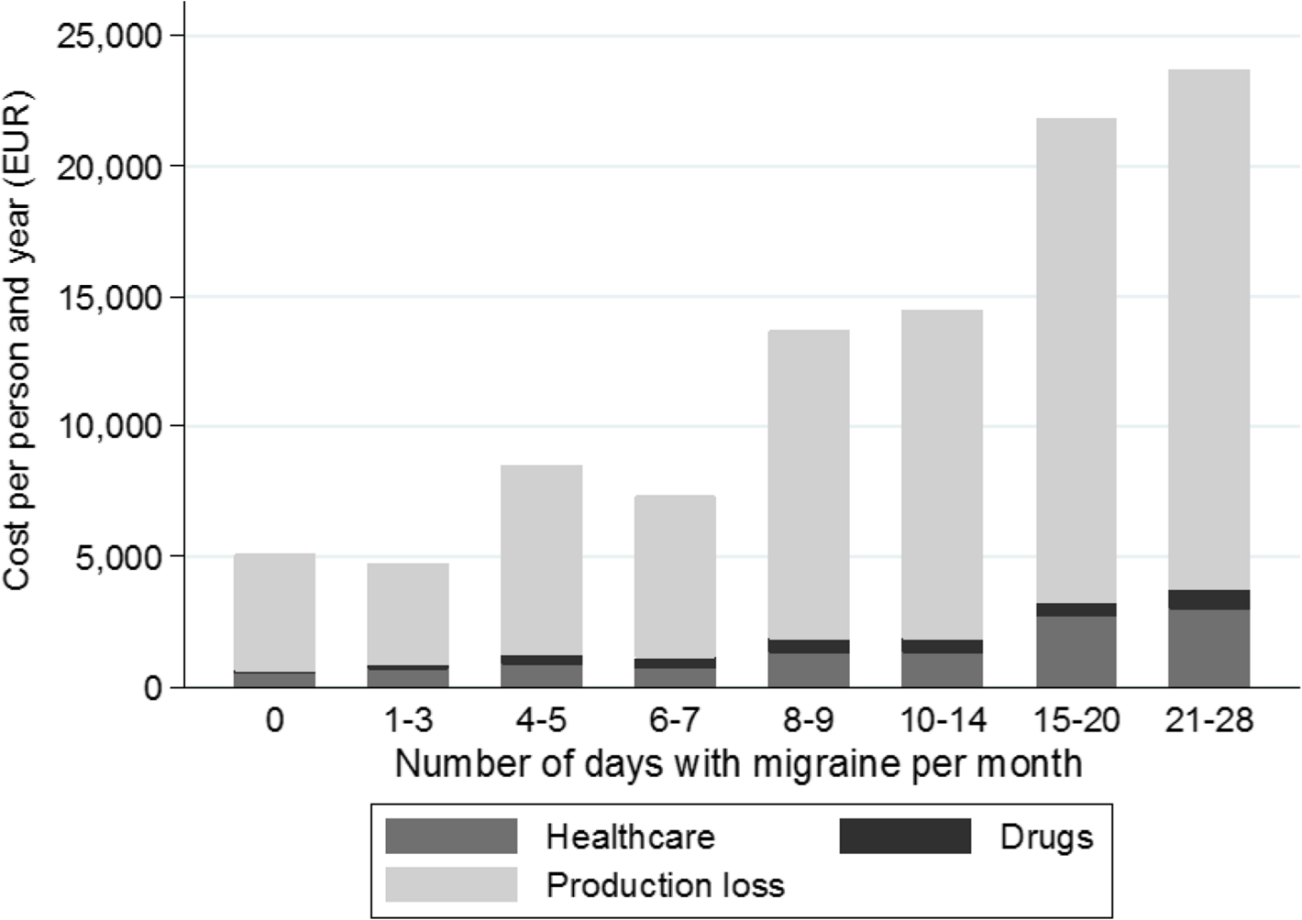
Average total cost (EUR) per person and year by number of migraine days per month

The total cost per patient and year increased with the number of migraine days per month (Fig. 3) and varied between €5000 and €24,000 per year. The average total cost per patient and year for all respondents amounts to €10,790. Indirect costs represented the main part of the costs, about 80%. The average total cost per patient and year for respondents with chronic migraine (defined as at least 15 headache days per month, whereof at least 8 days with migraine) was almost three times higher compared to the average total cost per patient and year for respondents with episodic migraine (€21,782 vs. €7598, *p* < 0.001). Both the number of headache and migraine days had a statistically significant impact on the total cost per person and year (*p* < 0.01). Other variables associated with total cost included age, employment, chronic migraine diagnosis and treatment with botulinum toxin (Additional file 1).

The difference in reported QoL between a day with and without migraine was 0.36 (*p* < 0.001) (Table 3). As showed in Table 3, the difference was less for those who responded to the questionnaire while having migraine (observable) compared to them who responded while not having migraine, thus recalling their health status the last day with migraine (retrospective).

**Table 3:** Mean quality of life a day with migraine and a day without migraine

	Day without migraine	Day with migraine	Difference	p-value^c^
EQ-5D				
All	0.786 (0.219)	0.422 (0.337)	0.364 (0.318)	< 0.001
	*N* = 630	*N* = 630		
Observable^a^	0.790 (0.190)	0.619 (0.229)	0.171	< 0.001
	*N* = 454	*N* = 176		
Retrospective^b^	0.775 (0.253)	0.346 (0.341)	0.429	< 0.001
	*N* = 176	*N* = 454		
EQ-VAS				
All	0.671 (0.209)	0.394 (0.210)	0.276 (0.248)	< 0.001
	*N* = 630	*N* = 630		
Observable ^a^	0.677 (0.189)	0.496 (0.215)	0.181	< 0.001
	*N* = 454	*N* = 176		
Retrospective ^b^	0.655 (0.253)	0.355 (0.240)	0.300	< 0.001
	*N* = 176	*N* = 454		

^a^Day without migraine = Respondents reporting not having migraine when completing the questionnaire. Day with migraine = Respondents reporting having migraine when completing questionnaire

^b^Day without migraine = Respondents reporting having migraine when completing questionnaire, asked to report for last day without migraine. Day with migraine = Respondents reporting not having migraine when completing the questionnaire, asked to report for last day with migraine

^c^T-test

**Fig. 4:**
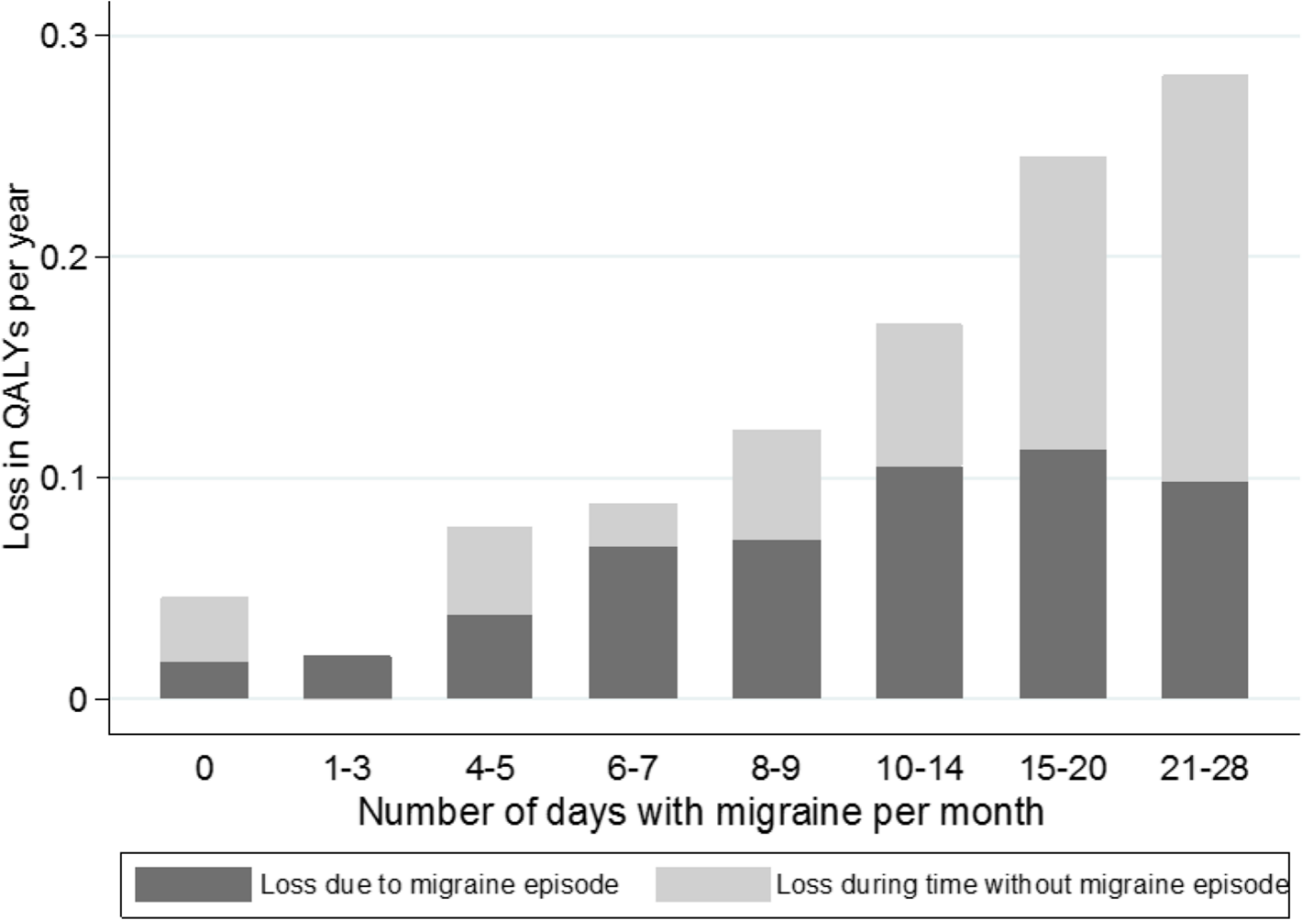
Average loss in QALYs per year and person by number of migraine days per month

The average loss in QALYs per person and years increased with the number of migraine days per month and varied between 0.019 and 0.259 QALYs (Fig. 4). The total average loss in QALYs per person and year was 0.10 and significantly higher for respondents with chronic migraine compared to respondents with episodic migraine (0.25 vs. 0.06, *p* < 0.001). The increase was most noticeable for the loss during the time without migraine episode (interictal state). The increase in the loss due to migraine episodes was limited because the baseline QoL (i.e. during a day without a migraine episode) for respondents with frequent migraine was already low. Both the number of headache and migraine days had a statistically significant impact on the loss in QALYs per person and year (*p* = 0.002, *p* = 0.023, data in Additional file 1).

## Discussion

This study shows that migraine leads to significant societal costs and loss of QoL. The average total cost per person and year was €10,790 and the average total QALY-loss per person and year was 0.10. The results of the analyses also show that both costs and loss in QoL increase with the number of headache and migraine days. Chronic migraine was associated with significantly higher cost, €21,782 vs. €7598, and QALY-loss, 0.25 vs. 0.06, compared to episodic migraine.

A major strength of the study is that long-term consequences of migraine in terms of long-term reduction of productivity and QoL is captured. To our knowledge it is also one of the first studies analysing the health-economic consequences of migraine for a Swedish context. The study differs from earlier studies considering health-economic aspects of migraine in that almost all respondents had been diagnosed by a doctor.

Almost all respondents had been diagnosed with migraine by a doctor according to their self-reported answer. A previous study showed that only about 50% of the respondents with self-reported migraine had received a diagnosis from a doctor in Sweden [3]. Although diagnosis frequency may have increased, the results of this study are likely not generalizable to the whole group of migraine patients. However, diagnosed patients are the primary candidates for new treatments and the results are therefore still relevant for future health economic analyses. The migraine diagnosis could not be checked or verified. However, provided that the respondents had joined a patient organization for headache disorders it is reasonable to assume that most of them had been diagnosed by a doctor. There may be additional selection bias since the sample was identified from a patient organization, which may include the more active participants and/or individuals with more severe migraine. The proportion of people with diagnosed CM or a migraine duration of at least 15 years was also found to be significantly higher than stated in other studies [3, 10]. The response rate may seem extremely low (22%). However, the questionnaire was sent to all members of Huvudvärksförbundet which includes individuals with different kinds of headache disorders. The real response rate could not be calculated since there were no information on the number of members with migraine.

This study is retrospective, i.e. respondents reported migraine experience, resource consumption and QoL for a period back in time. This implies a risk of recall bias which suggests caution in the interpretation of results. For example, in the QoL reporting there may be a recall bias in those with low frequency (e.g. if the last migraine day occurred some weeks before) as well as in those with high frequency (e.g. if the last day *without* migraine occurred weeks before). However, half of respondents reported that they used a migraine diary. Almost all reported at least one migraine day during the last 4 weeks. Information on headache and migraine frequency during the last 4 weeks was expanded to the entire year. This may cause some bias due to the migraine variability over time. For a majority (85%) the last 4 weeks had been as good or better than an average month, which means that the estimation is likely to be an underestimation.

There might also be a risk of bias in the QoL calculation as the reporting of both observable and retrospective EQ-5D might magnify the difference between the ictal and interictal phases, i.e. people might underestimate their QoL at the last attack compared to the current non-migraine day. The comparison of observable and retrospective responses demonstrated that observable responses gave a lower decrease in QoL. This result is consistent with the difference reported in other studies measuring QoL hypothetically (*ex ante*) [22] and observable (*ex post*) [24]. The hypothetical QoL is generally considered more relevant to decision making since it is assessed in a situation before suffering while the observable assessment implies an adaptation to the health state. The weights used for the baseline health state (assumed to correspond to age- and sex adjusted QoL reported for the Swedish general population) were different from the weights used for the ictal and interictal phases. This may have caused some bias in the estimation of the QALY loss during the time without a migraine episode (interictal phases). Since there are no studies using EQ-5D-5 L for the Swedish general population, it is difficult to estimate to what extent this is a source of bias. However, the utility weights used were relatively high (between 0.74 and 0.91) and studies comparing different types of weights do not find much differences at this level [25].

In our study, the annual healthcare cost of CM was estimated to around € 3668 per person and the annual cost of episodic migraine was estimated to approximately € 1331. The results are in the upper range of results reported in a previous European study by Bloudek et al. 2012, covering data from the UK, France, Germany, Italy and Spain [9], which might be explained by our study almost only including persons with a physician’s diagnosis, while Bloudek et al. included a large amount of self-diagnosed persons.

The total societal cost in our study amounted to approximately € 10,790 per person and year, i.e. almost ten times higher than reported in another European study Linde et al. 2012 covering eight different countries (Austria, France, Germany, Italy, Lithuania, Luxembourg, Netherlands and Spain), € 1222 per person [10]. One reason for this substantial difference may be that both our and the study by Bloudek et al. included more respondents with a more severe type of migraine. Our study addressed members of the patient association, Bloudek et al. addressed a stratified sample of an internet panel who suffered from headaches while Linde et al. targeted people with self-reported migraine in samples of the general population.

The loss in QoL related to a migraine episode has previously been investigated with EQ-5D in the United States (US) [15] and the United Kingdom (UK) [13]. The US study allowed respondents to complete the EQ-5D during a migraine episode and a day after treatment initiation. The loss in QoL was 0.14 for those with mild migraine, 0.19 for those with moderate migraine and 0.49 for those with severe migraine. In the UK study, people with migraine retrospectively completed EQ-5D for different levels of severity during one single migraine episode resulting in a loss in QoL of 0.21 for mild migraine, 0.34 for moderate migraine and 1.07 for severe migraine. In our study, the loss in QoL was estimated to 0.36. Based on HIT-6, the people who answered in our study had a severe form of migraine. This loss in QoL is lower than in the US and the UK. For the US study, this might be explained by the fact that the respondents assessed their QoL 1 day after initiation of treatment. Possibly, the QoL is then perceived higher than in general due to the current pain relief. In the UK study, the respondents assessed their QoL at a time of the migraine episode when feeling the worst. In our study, respondents reported QoL during a whole migraine episode. Comparisons between countries should however be interpreted with caution as they often differ due to other reasons.

## Conclusions

This study shows that migraine represents a significant burden on society and the individual and that there is a need for new and better treatment options in migraine. Both costs and loss in quality of life increases with the number of migraine days. Thus, there is potential for both cost savings and quality of life benefits connected with a reduction in the number of headache and migraine days.

## Additional file

Additional file 1: **Table S1.** Mean cost (SEK) per patient and year. **Table S2.** Average loss in QALY per person and year. **Table S3.** Regression of total cost per person and year. **Table S4.** Regression of total loss in QALY per person and year. (DOCX 32 kb)

## Abbreviations

CM: Chronic migraine
COI: Cost-of-illness
EQ-5D: Euro-Qol 5 dimensions
FASS: Pharmaceutical specialties in Sweden
HIT-6: Headache impact test
NSAID: Non-steroidal anti-inflammatory drugs
QALY: Quality adjusted life years
QoL: Quality of life

## Footnotes

1 No migraine during the last 4 weeks, but migraine reported during the last year.
