# Supplementary material for: Burden and costs of migraine in a Swedish defined patient population – a questionnaire-based study
Source: J Headache Pain. 2019 May 31;20(1):65. doi: 10.1186/s10194-019-1015-y (PMC6734313; doi:10.1186/s10194-019-1015-y)
Supplement: Supplementary file 1 — Table S1. Mean cost (SEK) per patient and year. Table S2. Average loss in QALY per person and year. Table S3. Regression of total cost per person and year. Table S4. Regression of total loss in QALY per person and year. (DOCX 32 kb) [file 10194_2019_1015_MOESM1_ESM.docx]

**Additional file 1**

**Table S1.** Mean cost (SEK) per patient and year

|  | **0 (n=43)** |  | **1-3 (n=155)** |  | **4-5 (n=122)** |  | **6-7 (n=73)** |  | **8-9 (n=60)** |  | **10-14 (n=95)** |  | **15-20 (n=55)** |  | **21-28 (n=27)** |  | **All (n=630)** | |
| --- | --- | --- | --- | --- | --- | --- | --- | --- | --- | --- | --- | --- | --- | --- | --- | --- | --- | --- |
|  | **Mean** | **Std. Dev.** | **Mean** | **Std. Dev.** | **Mean** | **Std. Dev.** | **Mean** | **Std. Dev.** | **Mean** | **Std. Dev.** | **Mean** | **Std. Dev.** | **Mean** | **Std. Dev.** | **Mean** | **Std. Dev.** | **Mean** | **Std. Dev.** |
|  |  |  |  |  |  |  |  |  |  |  |  |  |  |  |  |  |  |  |
| Emergency room or inpatient care | 171 | 782 | 767 | 3 810 | 718 | 3 107 | 151 | 734 | 400 | 2 660 | 1 204 | 6 881 | 9 399 | 45 180 | 7 689 | 21 558 | 1 727 | 14 679 |
| Visit neurologist | 2 292 | 4 215 | 1 926 | 4 687 | 2 937 | 5 193 | 4 050 | 5 228 | 5 574 | 7 365 | 5 532 | 6 775 | 6 298 | 7 956 | 8 737 | 8 111 | 3 958 | 6 203 |
| Other visits | 4 804 | 6 497 | 6 476 | 9 540 | 8 517 | 12 026 | 7 656 | 7 233 | 14 037 | 21 122 | 13 220 | 15 385 | 19 483 | 23 612 | 23 424 | 21 547 | 10 493 | 15 087 |
| Examinations | 583 | 1 491 | 607 | 1 627 | 758 | 1 749 | 487 | 1 107 | 415 | 1 002 | 692 | 2 363 | 1 487 | 2 250 | 2 668 | 4 292 | 780 | 1 970 |
| Botulinumtoxin type A | 868 | 2 868 | 996 | 2 859 | 2 652 | 4 385 | 2 830 | 4 644 | 4 314 | 5 242 | 3 563 | 4 960 | 3 982 | 4 732 | 4 701 | 5 317 | 2 643 | 4 437 |
| Acute drugs | 394 | 612 | 407 | 609 | 625 | 684 | 934 | 1 053 | 844 | 796 | 1 337 | 1 520 | 1 949 | 2 041 | 2 339 | 2 530 | 909 | 1 291 |
| Preventive drugs | 209 | 556 | 200 | 385 | 281 | 695 | 286 | 491 | 222 | 379 | 418 | 846 | 265 | 478 | 545 | 652 | 282 | 586 |
| **TOTAL HEALTH CARE COSTS** | **9 321** | **13 520** | **11 380** | **16 320** | **16 489** | **19 325** | **16 393** | **15 442** | **25 806** | **30 447** | **25 966** | **26 891** | **42 863** | **72 274** | **50 102** | **41 541** | **20 791** | **31 842** |
|  |  |  |  |  |  |  |  |  |  |  |  |  |  |  |  |  |  |  |
| Short-term sick leave | 6 399 | 16 573 | 17 375 | 42 527 | 28 280 | 63 104 | 31 549 | 62 799 | 30 959 | 68 680 | 60 516 | 115 818 | 57 400 | 103 439 | 53 579 | 130 076 | 33 225 | 77 728 |
| Reduced presenteeism | 21 663 | 67 895 | 11 754 | 18 290 | 34 540 | 65 832 | 32 527 | 39 386 | 28 733 | 39 061 | 42 195 | 49 191 | 68 560 | 77 734 | 27 937 | 46 105 | 31 110 | 52 492 |
| Long-term sick leave | 21 928 | 101 116 | 14 566 | 75 383 | 18 916 | 80 417 | 5 662 | 28 548 | 73 528 | 135 549 | 39 212 | 106 196 | 82 438 | 150 007 | 143 351 | 187 030 | 35 656 | 106 335 |
| **TOTAL PRODUCTIONLOSS** | 49 990 | 119 977 | 43 696 | 99 193 | 81 735 | 127 044 | 69 738 | 76 783 | 133 220 | 148 662 | 141 923 | 164 164 | 208 398 | 176 345 | 224 867 | 201 991 | 99 991 | 144 213 |
|  |  |  |  |  |  |  |  |  |  |  |  |  |  |  |  |  |  |  |
| **TOTAL COST** | 59 311 | 127 031 | 55 075 | 103 271 | 98 224 | 135 454 | 86 131 | 83 566 | 159 026 | 168 489 | 167 889 | 176 071 | 251 261 | 190 665 | 274 969 | 229 618 | 120 782 | 158 612 |

**Table S2.** Average loss in QALY per person and year

| **Number of migraine days** | **0 (n=43)*** |  | **1-3 (n=155)** |  | **4-5 (n=122)** |  | **6-7 (n=73)** |  | **8-9 (n=60)** |  | **10-14 (n=95)** |  | **15-20 (n=55)** |  | **21-28 (n=27)*** |  | **All (n=630)** |  |
| --- | --- | --- | --- | --- | --- | --- | --- | --- | --- | --- | --- | --- | --- | --- | --- | --- | --- | --- |
|  | **Mean** | **Std. Dev.** | **Mean** | **Std. Dev.** | **Mean** | **Std. Dev.** | **Mean** | **Std. Dev.** | **Mean** | **Std. Dev.** | **Mean** | **Std. Dev.** | **Mean** | **Std. Dev.** | **Mean** | **Std. Dev.** | **Mean** | **Std.Dev.** |
|  |  |  |  |  |  |  |  |  |  |  |  |  |  |  |  |  |  |  |
| QALY-loss, migraine episode | 0.017 | 0.067 | 0.019 | 0.025 | 0.038 | 0.046 | 0.069 | 0.066 | 0.072 | 0.076 | 0.105 | 0.112 | 0.113 | 0.150 | 0.099 | 0.162 | 0.058 | 0.090 |
| QALY-loss without migraine episode | 0.029 | 0.235 | -0.001 | 0.173 | 0.040 | 0.182 | 0.020 | 0.176 | 0.049 | 0.184 | 0.064 | 0.234 | 0.132 | 0.291 | 0.183 | 0.330 | 0.046 | 0.215 |
| **TOTAL LOSS IN QALYs** | **0.037** | **0.225** | **0.019** | **0.170** | **0.077** | **0.180** | **0.088** | **0.184** | **0.121** | **0.183** | **0.169** | **0.254** | **0.245** | **0.263** | **0.259** | **0.309** | **0.102** | **0.221** |

**Table S3.** Regression of total cost per person and year

| VARIABLES | Ln(totalcost) per person and year |
| --- | --- |
|  |  |
| Age | -0.0144** |
|  | (0.00597) |
| Woman=1 | 0.0375 |
|  | (0.174) |
| Employed=1 | 1.750*** |
|  | (0.143) |
| University education=1 | 0.0641 |
|  | (0.120) |
| Children in household=1 | 0.305** |
|  | (0.130) |
| >1 adult in household=1 | -0.00331 |
|  | (0.130) |
| Treatment with botulinumtoxin=1 | 0.395*** |
|  | (0.146) |
| Time with migraine diagnosis | -0.00376 |
|  | (0.00823) |
| Chronic migraine diagnosis by doctor=1 | 0.451*** |
|  | (0.144) |
| Chronic migraine based on number of reported days with migraine | -0.251 |
|  | (0.229) |
| Number of headache days | 0.0363*** |
|  | (0.0109) |
| Number of migraine days | 0.0806*** |
|  | (0.0154) |
| Comorbidity=1 | 0.218* |
|  | (0.116) |
| Constant | 8.513*** |
|  | (0.440) |
|  |  |
| Observations | 587 |
| R-squared | 0.454 |
| Standard errors in parentheses |  |
| *** p<0.01, ** p<0.05, * p<0.1 |  |

**Table S4.** Regression of total loss in QALY per person and year

| VARIABLES | Total QALY-loss per person and year |
| --- | --- |
|  |  |
| Age | -0.00450*** |
|  | (0.000841) |
| Woman | -0.0162 |
|  | (0.0245) |
| Employed=1 | -0.0430** |
|  | (0.0201) |
| University education=1 | -0.0285* |
|  | (0.0168) |
| Children in household=1 | 0.00846 |
|  | (0.0183) |
| >1 adult in household=1 | -0.0192 |
|  | (0.0184) |
| Treatment with botuliniumtoxin =1 | 0.0139 |
|  | (0.0205) |
| Time with migraine diagnosis | 0.000125 |
|  | (0.00116) |
| Chronic migraine diagnosis by doctor=1 | 0.0107 |
|  | (0.0201) |
| Chronic migraine based on number of reported days with migraine =1 | 0.0396 |
|  | (0.0323) |
| Number of headachedays | 0.00471*** |
|  | (0.00154) |
| Number of migraine days | 0.00495** |
|  | (0.00217) |
| Comorbidity=1 | 0.0811*** |
|  | (0.0163) |
| Constant | 0.245*** |
|  | (0.0621) |
|  |  |
| Observations | 593 |
| R-squared | 0.262 |
| Standard errors in parentheses | |
| *** p<0.01, ** p<0.05, * p<0.1 | |
